# Speech, stone tool-making and the evolution of language

**DOI:** 10.1371/journal.pone.0191071

**Published:** 2018-01-19

**Authors:** Dana Michelle Cataldo, Andrea Bamberg Migliano, Lucio Vinicius

**Affiliations:** Department of Anthropology, University College London, London, United Kingdom; Massachusetts Institute of Technology, UNITED STATES

## Abstract

The ‘technological hypothesis’ proposes that gestural language evolved in early hominins to enable the cultural transmission of stone tool-making skills, with speech appearing later in response to the complex lithic industries of more recent hominins. However, no flintknapping study has assessed the efficiency of speech alone (unassisted by gesture) as a tool-making transmission aid. Here we show that subjects instructed by speech alone underperform in stone tool-making experiments in comparison to subjects instructed through either gesture alone or ‘full language’ (gesture plus speech), and also report lower satisfaction with their received instruction. The results provide evidence that gesture was likely to be selected over speech as a teaching aid in the earliest hominin tool-makers; that speech could not have replaced gesturing as a tool-making teaching aid in later hominins, possibly explaining the functional retention of gesturing in the full language of modern humans; and that speech may have evolved for reasons unrelated to tool-making. We conclude that speech is unlikely to have evolved as tool-making teaching aid superior to gesture, as claimed by the technological hypothesis, and therefore alternative views should be considered. For example, gestural language may have evolved to enable tool-making in earlier hominins, while speech may have later emerged as a response to increased trade and more complex inter- and intra-group interactions in Middle Pleistocene ancestors of Neanderthals and *Homo sapiens*; or gesture and speech may have evolved in parallel rather than in sequence.

## Introduction

According to the ‘technological hypothesis’, even the manufacture of Oldowan (Mode 1) simple artefacts may have required the cultural transmission of tool-making skills [1–7]. Since tool-making is a hand-based activity, gestural teaching would be originally selected over vocalisations as a teaching aid [8, 9]. It follows from the technological hypothesis that speech was a later addition to human language, but how stone tool-making relates to speech evolution remains unclear. Brain imaging studies have revealed functional and anatomical associations between hand, oral and language cortical areas [10], suggesting that language production could have migrated from hand to mouth [11–13]. A few experimental flintknapping studies have investigated the possibility that speech evolved to enable the teaching of more complex tool-making skills. Morgan and collaborators [14] compared the transmission efficiency of Oldowan tool-making skills from trained tutors to subjects under five communication treatments: reverse engineering (asocial learning), emulation (social learning without teaching), basic teaching (rudimentary visual communication), gestural language (gesture-assisted teaching), and ‘full language’ (gesture plus speech; sample size was 6 to 8 tutor-to-apprentice transmission trials in each treatment). Transmission efficiency increased across treatments from reverse engineering to full language, although full language was not significantly superior to gestural language in any of six measures of tool-making performance. The conclusion was that gestural language probably evolved before speech as a result of selective pressures generated by cultural transmission demands emerging with the simple Oldowan industry. Speech would have evolved later to support more sophisticated, dynamical and diversified lithic industries associated with more recent hominins.

However, flintknapping studies focusing on later lithic industries have not been able to demonstrate a superiority of vocal communication over gesture as a teaching aid. Putt and collaborators [15] compared transmission of Acheulean (Mode 2) tool-making skills under two treatments: a verbal group (transmission ‘via spoken communication, and by example’; *N* = 13 individuals), and a nonverbal or gestural group (*N* = 11). They found no differences in three measures of Acheulean handaxe quality, although the verbal group produced worse flakes possibly due to a tendency to overimitate demonstrators. Ohnuma and colleagues [16] focused on the experimental production of Levallois flakes (Mode 3) characteristic of the African Middle Stone Age and the European Middle Palaeolithic. There were no differences either in skill acquisition or flake quality between a ‘gesture alone’ group (*N* = 10) and a ‘verbal’ group relying on ‘spoken language and visual demonstration’ (*N* = 10), despite the fact that only the verbal group included senior students with prior knowledge of lithic technologies. Thus, the relationships among speech evolution, cultural transmission and lithic industries still require clarification.

While previous flintknapping studies have compared gesture alone to gesture plus speech (full language), with the latter alternatively defined as ‘speech and visual demonstration’ [16], ‘spoken communication and example’ [15], or gestural teaching plus speech [14], remarkably they have never analysed speech alone as an aid to cultural transmission of tool-making skills. The distinction between speech and gesture in experimental approaches may provide important insights into language origins, reveal possibly distinct selective pressures behind language evolution, and enable comparisons between the technological hypothesis and other theories. For example, social models of language origins propose that although gestural communication in earlier hominins may have performed imperative, demonstrative and expressive functions, human language only evolved with the emergence of vocal communication, social gossip, grammar and recursive mindreading [17–19]. Other authors believe that human language and speech emerged less than 100,000 years ago [20], a scenario radically dissociating language origins from early hominin tool-making.

Here we investigate the performance of speech as a teaching aid during experimental transmission of Oldowan-style tool-making skills. By comparing the flintknapping performance of subjects instructed through gesture alone, speech alone, full language (gesture plus speech) as well as a control (no instruction) group, we show that speech is an inferior teaching aid compared to both gesture and full language according to four estimators of stone tool-making skill. Questionnaires also revealed that subjects receiving verbal training alone were significantly less satisfied than the group instructed by gesture. The low efficiency of speech to support the transmission of even the simplest Oldowan techniques may explain why gesture was likely to be selected over speech as a teaching aid in early hominins, and why full language (rather than speech alone) became the default communication mode of *Homo sapiens*. Our results suggest that speech did not evolve as a superior method of cultural transmission of tool-making skills, and possibly evolved for reasons other than assisting lithic production. Therefore, we may need to consider alternatives to the technological hypothesis. For example, gestural language may have originally emerged to enable the cultural transmission of tool-making skills in early hominins, while speech and grammar may have later evolved as a result of increased inter-group interactions and long-distance trade in Middle Pleistocene ancestors to Neanderthals and modern humans; or gesture and speech may have emerged in parallel rather than in sequence, reflecting possibly distinct communicative functions.

## Results

### Speech is an ineffective method of transmission of Oldowan-style tool-making skills

**Fig 1:**
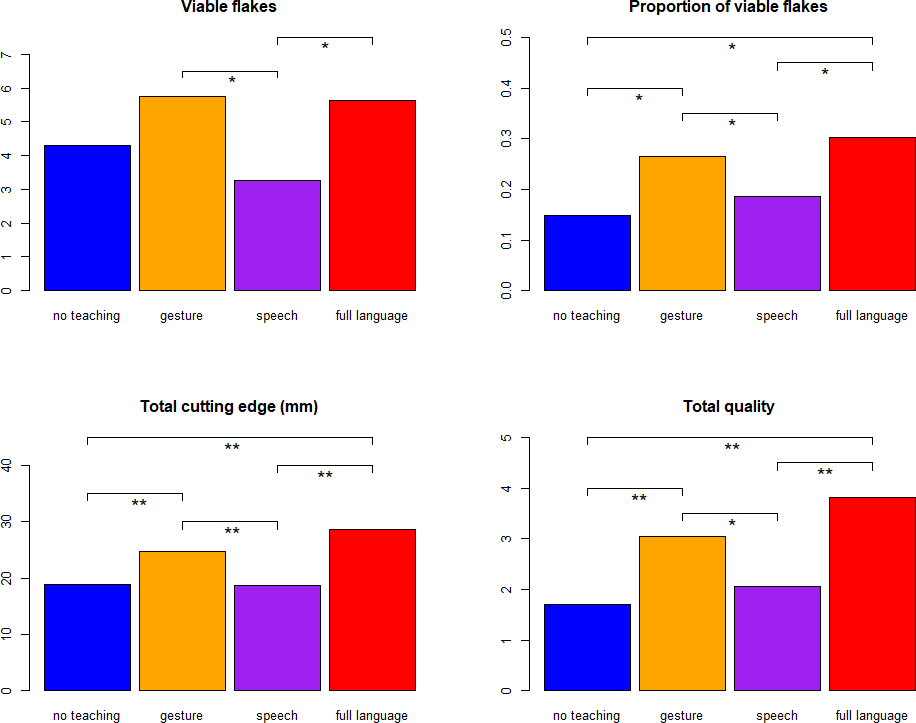
Flintknapping performance as a function of communication group. Measures of performance on the y-axis are number of produced viable flakes, proportion of viable flakes over total flakes, cutting edge of flakes (mm), and total flake quality. Data from 71 participants split into four communication conditions: no teaching (*N* = 10), gesture (*N* = 27), speech (*N* = 23) and full language (*N* = 11). Total cutting edge and total quality were measured for each viable flake and averaged by participant. The four communication groups were compared through pairwise Wilcoxon rank sum tests with fdr (false discovery rate) correction for multiple testing. Significant differences represented by horizontal bars. Significance levels: *P*<0.05 (*) and *P*<0.01 (**).

Flintknapping experiments with a total of 71 participants revealed significant differences across the four communication groups (no instruction: *N* = 10; gesture: *N* = 27; speech: *N* = 23; full language: *N* = 11) in four measures of flintknapping performance: number of produced viable flakes (Kruskal-Wallis rank sum test, χ^2^ = 10.2, df = 3, *P* = 0.017), proportion of viable flakes (χ^2^ = 13.5, df = 3, P = 0.0036), total flake cutting edge (χ^2^ = 19.5, df = 3, *P*<0.001), and total flake quality (χ^2^ = 21.9, df = 3, *P*<0.001; see Materials and Methods for experimental procedures, variable definitions and statistical analyses). Despite all its grammatical and semantic resources, speech was an inferior instruction aid in comparison to gesture according to all four measures of skill (Fig 1; see Table 1 for descriptive statistics, and S1 Table for pairwise Wilcoxon tests). The speech group also performed significantly worse than the full language group in all four measures, and as poorly as the control group receiving no instruction (no significant differences in any of the four measures). The full language and gesture groups did not differ from each other in any of the measures. In summary, speech is an inefficient means of transmission of Oldowan tool-making skills in comparison to gesture, implying that the high efficiency of full language seems to be a consequence of its gestural rather than vocal component.

**Table 1:** Means (standard deviations) and medians (ranges) of flake measurements by communication group.

Variable		No instruction (*N* = 10)	Gesture (*N* = 27)	Speech (*N* = 23)	Full language (*N* = 11)
**Number of viable flakes**		4.3(4.2), 3(1–15)	**5.8**(5.9), 4(1–32)	3.3(2.9), 3(0–11)	5.6(2.4), **7**(2–10)
**Proportion of viable flakes**		0.15(0.1), 0.11(0.04–0.37)	0.27(0.13), 0.24(0.08–0.55)	0.19(0.16), 0.13(0–0.67)	**0.3**(0.11), **0.3**(0.14–0.5)
**Total cutting edge (mm)**		18.8(4.9), 19.2(11–25.3)	24.7(6.0), 23.4(11.7–41.6)	18.6(7.7), 20.6(0–27.8)	**28.7**(6.7), **28.5**(15–36)
**Total flake quality**		1.7(0.53), 1.7(0.83–2.6)	3(1.1), 3(1.4–6.2).	2.1(1.2), 2.4(0–3.9)	**3.8**(1.2), **4**(1.6–5.6)
**Scores**	**Question 1**	1.7(0.82), 1.5(1–3)	3.5(1.1), **4**(1–5)	2.8(1.1), 3(1–5)	**3.5**(0.82), **4**(2–5)
	**Question 2**	1.8(0.42), 2(1–2)	3.3(1.1), **4**(1–5)	2.3(1.3), 2(1–5)	**4.0**(0.9), **4**(2–5)
	**Question 3**	1.5(0.53), 1.5(1–2)	3.5(1.0), **4**(1–5)	2.6(1.3), 2(1–5)	**3.8**(0.87), **4**(2–5)

Total sample: *N* = 71 participants. Highest mean and median values of each measurement or question score are shown in bold.

### Subjects rate speech as an inefficient tool-making teaching aid

**Fig 2:**
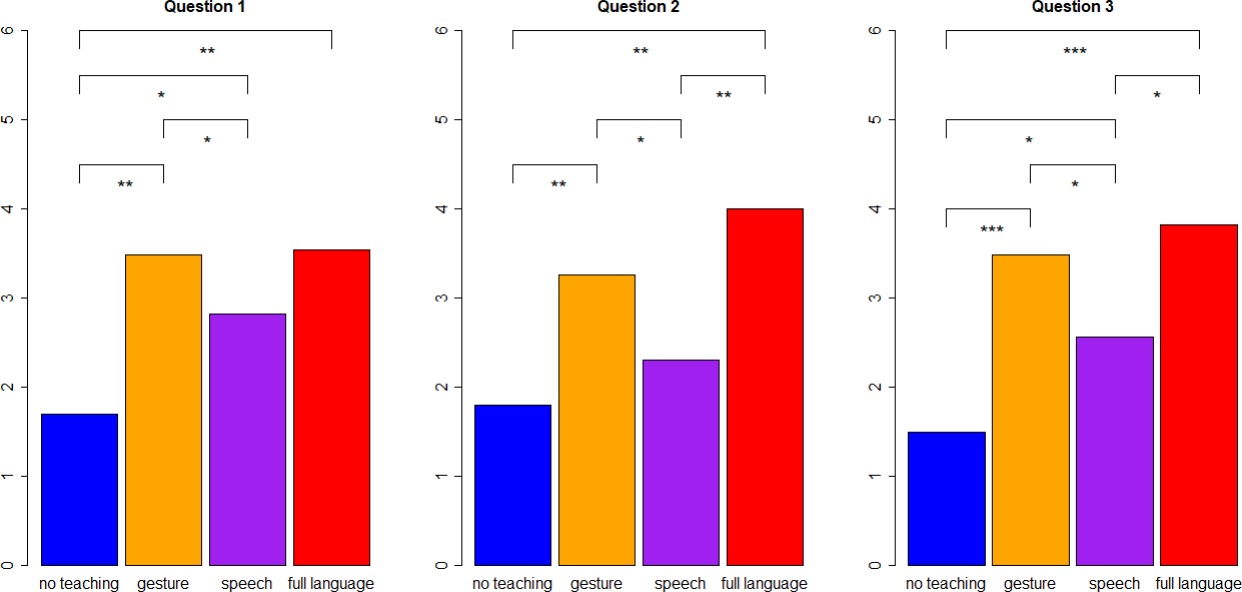
Satisfaction of participants with received instruction as a function of communication treatment. Y-axis values represent mean agreement of participants (measured on a scale from 1 to 5) with three statements about their received instruction (Questions 1 to 3). Data from 71 participants split into four communication conditions: no teaching (*N* = 10), gesture (*N* = 27), speech (*N* = 23) and full language (*N* = 11). The four communication groups were compared through pairwise Wilcoxon rank sum tests with fdr (false discovery rate) correction for multiple testing. Significant differences represented by horizontal bars. Significance levels: *P*<0.05 (*), *P*<0.01 (**) and *P*<0.001 (***).

Satisfaction was measured through agreement scores on a 1-to-5 scale to three questions, and significantly differed across the four treatment groups (Kruskal-Wallis rank sum test; Question 1; χ^2^ = 19.2, df = 3, *P*<0.001; Question 2: χ^2^ = 22.3, df = 3, *P*<0.001; Question 3: χ^2^ = 24.8, df = 3, *P*<0.001; see Materials and Methods for details of questionnaire). Speech consistently ranked lower than gesture as a teaching aid (Fig 2 and S1 Table). The speech group also showed significantly lower satisfaction scores than the full language group in two of the three questions. Participants instructed through speech showed mean and median satisfaction scores below the mid-point of 3 on the 1-to-5 scale, with the exception of Question 3 with median at the mid-point (Table 1). The speech group was significantly more satisfied than the control group receiving no instruction according to two of the three questions, although their tool-making performance was overall similar. Instruction via full language and gesture received the highest ratings (with medians and means consistently above the mid-point on the satisfaction scale), which did not significantly differ between the two groups. Therefore, adding verbal communication to gestural teaching (in the full language group) increased neither flintknapping performance nor satisfaction with received instruction in comparison to instruction by gesture alone.

## Discussion

Our experiments and questionnaire provide convincing evidence that speech is an inefficient assistant to the cultural transmission of Oldoway-style tool-making skills. Earlier hominins were cognitively and culturally distinct from modern humans, and speech in its present form is likely to be significantly more effective as a communicative tool than near its origin. Modern humans also heavily depend on speech, and for this reason we might have expected a bias towards speech over gesture in a cultural transmission experiment. Nonetheless, our experiments provide experimental support to the idea that the evolution of gestural language preceded speech due to its superiority as a tool-making teaching aid. Since subjects instructed exclusively through vocal communication performed almost as poorly as control groups receiving no training, we found no evidence that speech could have evolved to enable the transmission of stone tool-making knowledge in earlier hominins.

The lower efficiency of speech in assisting lithic production leaves us with the question of why vocal communication evolved at all. Corballis [21] proposed that language first emerged as gesture, with speech later evolving to release hands from the multiple burdens of tool-making and communication. Speech would result in a teaching improvement since ‘vocal language allows people to use tools and at the same time explain verbally what they are doing, leading perhaps to pedagogy’. However, according to our results speech was unlikely to engender superior pedagogy either alone or even as a component of full language, which did not exhibit significantly higher transmission efficiency than gesture alone. Another proposal was that speech was a requirement for the origin of more sophisticated and dynamical technologies emerging after Oldowan or Acheulean industries [14]. Testing this idea requires a not yet attempted comparison of speech alone, gesture alone and full language as teaching aids to cultural transmission of post-Oldowan lithic techniques. However, the flintknapping studies of Acheulean and Levallois tool-making reviewed above did not reveal any superiority of full language over gesture [15, 16], suggesting that the same may be true for speech alone. Furthermore, the relative inefficiency of speech in tool-making skill transmission may also explain why it has not fully replaced gesture as the default communication mode of *Homo sapiens*. Instead, gestural communication was preserved as a key functional assistant to speech in our current full language [22, 23] even in congenitally blind people [24]. It could also be argued that teaching via speech would be advantageous to earlier tool-makers under nocturnal or suboptimal visual conditions. However, this idea has little empirical support. Hunter-gatherers for example predominantly produce and use tools during the day, and their conversations almost never relate to technical knowledge, especially in the evenings; the Ju’/hoansi (!Kung) Bushman for instance dedicate 80% of their evening conversations to social topics and storytelling [25].

We conclude that flintknapping studies have not yet provided definitive evidence that speech evolved to enable the cultural transmission of either simple or complex tool-making techniques. Therefore, we cannot discard the possibility that speech may have evolved for reasons other than lithic technology, or even that gesture and speech may have evolved in parallel rather than in sequence. As a result, we may need to consider alternatives to the technological hypothesis. For example, one possibility is that gestural language may have evolved to support the cultural transmission of the first lithic technologies, while speech and grammar would have later evolved to assist in the transmission of other types of cultural information, or to mediate new forms of social interaction. Gestural language would have been retained alongside speech as a component of full language due to its role in transmission of tool-making skills. This scenario is compatible with recent findings in neuroscience, genetics and archaeology. For example, although it is widely believed that humans possess a uniquely derived vocal apparatus compared to other primates in general and apes in particular [26], the vocal tract of macaques seem to be ‘speech-ready’ or able to produce most speech sounds associated with human spoken language [27]. The human superiority in vocal production may therefore be explained by uniquely sophisticated neural control rather than special vocal anatomy. The *FOXP2* gene is known to regulate a developmental pathway responsible for speech control (among other linguistic and non-linguistic functions), with human mutations causing severe deficits in speech production due to defective neural control of orofacial movements rather than abnormal vocal tract anatomy [28]. The two aminoacid substitutions differentiating human *FOXP2* from that of other primates were also found in Neanderthals and reveal signs of positive selection probably related to speech evolution, with a selective sweep starting in the common ancestor of humans and Neanderthals around 400,000–300,000 years ago, and fixation of substitutions occurring within the last 260,000 years [29]. This timeframe roughly overlaps with significant changes in hominin social organisation. A recent study of a site representative of the later Middle Pleistocene in East Africa (400,000–130,000 years ago) revealed Levallois artefacts dating to at least 200,000 years ago and mostly derived from an obsidian source over 160 km away [30]. This provides further evidence of increase long-distance raw material transport, trade expansion, and possible intensification of interactions among geographically distinct human populations. Such sociodemographic changes and more complex inter- and intra-group interactions may have set the selective context for the origin of speech in Middle Pleistocene hominins ancestral to Neanderthals and *Homo sapiens*. Another possibility is that gesture and speech evolved in parallel in early hominins, with gesture assisting in tool-making, and speech contributing to other dimensions of hominin life such as social interactions or coordination of cooperative activities, or providing an alternative to gesture under suboptimal visual conditions.

Our study has presented an experimental rejection of a direct link between speech and the teaching of tool-making skills, which can arguably be described as negative evidence. This points to the need of further studies to establish positive evidence for speech evolution, namely the experimental investigation (along the lines of the lab tests we presented above) of alternative selective pressures potentially explaining why speech evolved. For example, new experiments should examine the efficiency of speech alone as a teaching medium of later lithic technologies (from Acheulean up to sophisticated microlithic, Mode 5 industries); measure the performance of speech in assisting the cultural transmission of non-lithic skills; or compare gestural language to a simplified or ungrammatical ‘proto-speech’ in experiments focusing on the transmission of social rather than cultural information. In summary, future studies should contemplate the possibility that human language, which incorporate both gesture and speech, may be the result of multiple selective pressures including but not limited to the cultural transmission of tool-making skills.

## Materials and methods

### Ethics statement

Our study was registered with the University College London Data Protection Office and exempt from approval by the University College London Ethics Committee (as it only involved non-sensitive and anonymised tests on non-vulnerable participants). All subjects read a leaflet describing the purposes and outcomes of the experiments and signed consent forms.

### Experimental design

The study was based on the hypothesis that instruction by either speech alone or gesture alone would have a significant effect on flintknapping performance of human subjects. A no-instruction group was included as a control, and a full language group (gesture plus speech) was added to represent the current mode of human communication. Testing of volunteers took place over three weeks to increase consistency of instruction (performed by one skilled tutor) and experimental conditions. Sample size was determined by the availability of volunteers during the recruitment stage. We aimed at randomly allocating twice as many individuals to speech and gesture groups (the main focus of our study) than to control and full language groups. Variation in group sizes also reflected no-shows by volunteers. One skilled flintknapping tutor was responsible for running the experimental sessions, assisted by the first author (DC). Each participant performed the experiment individually at an allocated time slot. Informed consent was obtained from all participants after they read an explanation of the study. All flake measurements were later processed by the first author (DC). Statistical analyses were performed by the corresponding author (LV). The following links provide footage of two sessions with examples respectively of the gesture (http://tinyurl.com/ojqaru9) and speech (http://tinyurl.com/nun34ty) treatments.

### Sample and materials

71 students from University College London and Cambridge University (38 men, 33 women; age range: 17–44 years) were assigned to four communication treatments: reverse engineering (no instruction; *N* = 10), gesture (*N* = 27), speech (*N* = 23), and full language (speech and gesture; *N* = 11). Experiments used 60 kg of Brandon flint and 37 hammerstones collected from Cromer Beach, Norfolk.

### Experimental flintknapping

In Phase 1 (Introduction, five minutes) participants received a sheet with basic information on flint knapping and Oldowan technology (but no instructions on flake production), a piece of chamois leather, wooden sticks, and flakes of variable quality. In Phase 2 (Teaching, five minutes), subjects received treatment-specific instructions on viable flake production by an experienced tutor (except for the control group). In Phase 3 (Flintknapping, 20 minutes) participants were asked to choose a hammerstone and flint core, and to produce as many viable flakes as possible. Participants were told to take as long as they wanted, but were stopped after 20 minutes. In Phase 4 (Classifying), participants were asked to classify their flakes into ‘viable’ and ‘not viable’. Detailed protocols are available on http://dx.doi.org/10.17504/protocols.io.jyycpxw.

### Questionnaires

After the experimental phases, participants ranked on a scale from 1 to 5 their agreement with three statements: 1) ‘The instruction received during the Teaching Phase of this experiment really helped me to produce high quality flakes’; 2) ‘The method of instruction I received during the Teaching Phase corresponds to how I learn best’; 3) ‘I believe that the instruction I was given during the Teaching Phase was effective in helping me to learn a mechanical task such as flint knapping’. The three statements are similar in content and had the common purpose of measuring satisfaction of subjects with their received training.

### Statistical analyses

Participants produced 1742 flakes and selected 542 as viable. Of those, only 335 were viable under the definition by Morgan and collaborators [14] requiring flakes to be greater than a threshold diameter. We assessed the 335 viable flakes through four measures of tool-making skill: number of viable flakes, proportion of viable flakes (viable flakes divided by produced flakes), length of flake cutting edge, and total flake quality, estimated by the formula [14]:

$$total\;flake\;quality=\frac{flake\;cutting\;edge}{\sqrt[{3}]{flake\;mass}}(1-e^{-0.31(flake\;diameter-1.81)})$$

Since the 335 flakes were produced by 71 participants, the four measures of skill were calculated (viable flakes, proportion of viable flakes) or averaged (flake cutting edge, total quality) for each subject. Based on individual values, we tested for differences across the four treatment groups through Kruskal-Wallis tests and pairwise Wilcoxon tests with correction for expected false discovery rate (fdr) in multiple testing. We used the same tests to compare satisfaction scores from our questionnaire. Non-parametric (rank) testing was applied throughout due to the small sample size of the control and full language groups, and also due to some variables not exhibiting normal distribution. The dataset included two outliers producing an exceptionally high number of viable flakes (one control, one in the gesture group). They were no outliers in the other three measures of skill. Elimination of the two outliers did not result in any change in the overall pattern of the results. Therefore, the final analyses included all 71 participants. See Supplementary Materials for detailed testing protocols and video links with examples of instruction sessions.

## Supporting information

S1 Table Pairwise Wilcoxon rank sum tests. Tested variables: viable flakes, proportion of viable flakes, total flake cutting edge, total flake quality. We also compared scores (on a scale from 1 to 5) measuring agreement of subjects to three statements about their satisfaction with received instruction (Questions 1, 2 and 3). Communication treatments: no instruction, gesture, speech, full language. Significant tests (*P*<0.05) highlighted in bold. Values corrected for false discovery rate (fdr) in multiple testing.
(DOCX)

S1 Dataset Excel file with full set of participant and flake data. (CSV)
