# Supplementary material for: Speech, stone tool-making and the evolution of language
Source: PLoS One. 2018 Jan 19;13(1):e0191071. doi: 10.1371/journal.pone.0191071 (PMC5774752; doi:10.1371/journal.pone.0191071)
Supplement: S1 Table — Tested variables: viable flakes, proportion of viable flakes, total flake cutting edge, total flake quality. We also compared scores (on a scale from 1 to 5) measuring agreement of subjects to three statements about their satisfaction with received instruction (Questions 1, 2 and 3). Communication treatments: no instruction, gesture, speech, full language. Significant tests (P<0.05) highlighted in bold. Values corrected for false discovery rate (fdr) in multiple testing. (DOCX) [file pone.0191071.s001.docx]

Speech, stone tool-making and the two-step evolution of language

Dana Michelle Cataldo, Andrea Bamberg Migliano, Lucio Vinicius

**S1 Table. Pairwise Wilcoxon rank sum tests**.

| Viable flakes | | | | Proportion of viable flakes | | | |
| --- | --- | --- | --- | --- | --- | --- | --- |
|  | Full language | Gesture | No instruction |  | Full language | Gesture | No instruction |
| Gesture | 0.365 |  |  | Gesture | 0.431 |  |  |
| No instruction | 0.162 | 0.365 |  | No instruction | **0.021** | **0.021** |  |
| Speech | **0.042** | **0.042** | 0.404 | Speech | **0.021** | **0.021** | 0.71 |
| Total cutting edge | | | | Total quality | | | |
|  | Full language | Gesture | No instruction |  | Full language | Gesture | No instruction |
| Gesture | 0.0735 |  |  | Gesture | 0.05376 |  |  |
| No instruction | **0.0046** | **0.008** |  | No instruction | **0.0011** | **0.0005** |  |
| Speech | **0.0046** | **0.0081** | 0.799 | Speech | **0.0027** | **0.024** | 0.318 |
| Question 1 | | | | Question 2 | | | |
|  | Full language | Gesture | No instruction |  | Full language | Gesture | No instruction |
| Gesture | 0.89 |  |  | Gesture | 0.061 |  |  |
| No instruction | **0.002** | **0.001** |  | No instruction | **0.001** | **0.0021** |  |
| Speech | **0.076** | **0.048** | **0.016** | Speech | **0.002** | **0.015** | **0.51** |
| Question 3 | | | |  | | | |
|  | Full language | Gesture | No instruction |  |  |  |  |
| Gesture | 0.367 |  |  |  |  |  |  |
| No instruction | **0.0005** | **0.0002** |  |  |  |  |  |
| Speech | **0.013** | **0.013** | **0.03** |  |  |  |  |

Tested variables: viable flakes, proportion of viable flakes, total flake cutting edge, total flake quality. We also compared scores (on a scale from 1 to 5) measuring agreement of subjects to three statements about their satisfaction with received instruction (Questions 1, 2 and 3). Communication treatments: no instruction, gesture, speech, full language. Significant tests (*P*<0.05) highlighted in bold. Values corrected for false discovery rate (fdr) in multiple testing.
